# Evaluation-independent system for DNA section amplification

**DOI:** 10.1186/s12938-018-0580-7

**Published:** 2018-11-06

**Authors:** Deuk-Ju Lee, Jong-Dae Kim, Yu-Seop Kim, Hye-Jeong Song, Chan-Young Park

**Affiliations:** 1 Department of Convergence Software, Hallym University, Chuncheon, South Korea; 2 Bio-IT Research Center, Hallym University, Chuncheon, South Korea

**Keywords:** Gel documentation system, Gel image analysis, DNA detection, Fluorescence, Optics, NTC thermistor, Thermocouple, Side illumination

## Abstract

**Background:**

In general, the image analysis of nucleic acid for detecting DNA is dependent on the gel documentation system. These experiments may deal with harmful staining agents and are time consuming. To address these issues, real-time polymerase chain reaction (PCR) devices have been developed. The advantages of real-time PCR are its capabilities for real-time diagnosis, improved sensitivity, and digitization of measurement results. However, real-time PCR equipment is still too bulky and expensive for use in small hospitals and laboratories.

**Methods:**

This paper describes an evaluation-independent real-time PCR system that differs from conventional systems in that it uses a side-illumination optical detection system and a temperature adjustment coefficient for DNA detection. The overall configuration of the evaluation-independent system includes the PCR chip and system hardware and software. The use of the side-illumination method for detection enables the system size to be reduced compared to systems using a typical illumination method. Furthermore, the results of a PCR test are strongly affected by the reaction temperature. Thus, extremely precise control of the temperature of the reaction is needed to obtain accurate results and good reliability. We derived a temperature compensation coefficient that allows us to compensate for the differences between the measured temperature of the negative temperature coefficient (NTC) thermistor sensor and the real temperature of the thermocouple.

**Results:**

Applying the temperature compensation coefficient parameter using the NTC thermistor and using the side-illumination method resulted in an increase in the initial sensor value. The occurrence of the DNA section amplification decreased to 22 cycles from 24 cycles.

**Conclusions:**

The proposed system showed comparable performance to that of an existing real-time PCR, even with the use of simpler and smaller optical devices.

## Background

Polymerase chain reaction (PCR) is an important technology used in biological research because it enables disease diagnoses with only a small amount of deoxyribonucleic acid (DNA) [1–3]. The DNA detection process requires four steps: DNA extraction, DNA amplification, electrophoresis, and gel image analysis [4–6]. A typical DNA detection method uses a gel documentation system. The PCR undergoes electrophoresis after amplification for the result analysis. Electrophoresis has a long processing time and uses EtBr, which is a carcinogen widely used for staining nucleic acid. Furthermore, its quantitative analysis is hindered by low sensitivity and poor resolution. Real-time PCR is currently used to address the drawbacks of the PCR. Real-time PCR technology enables the real-time monitoring of the amplification process for each cycle with a low contamination rate. The procedure also rapidly produces the analyzed results using the fluorescent detection technique [7, 8]. With these advantages, many studies have been conducted on the technique and various real-time PCR devices are available. However, real-time PCR equipment remains bulky and expensive, making it difficult to acquire and use for some laboratories. For example, real-time DNA amplification devices currently used in hospitals or research centers are too bulky to be easily moved. In our laboratory, the dimensions of the Bio-Rad CFX96 real-time PCR detection system are 33 cm × 46 cm × 36 cm. In this work, we propose a portable real-time PCR system that is much smaller. This system was manufactured in-house using commercially available parts and a self-made integration board and case. The methods, results, discussion, and conclusions are presented in the following sections.

## Methods

**Fig. 1:**
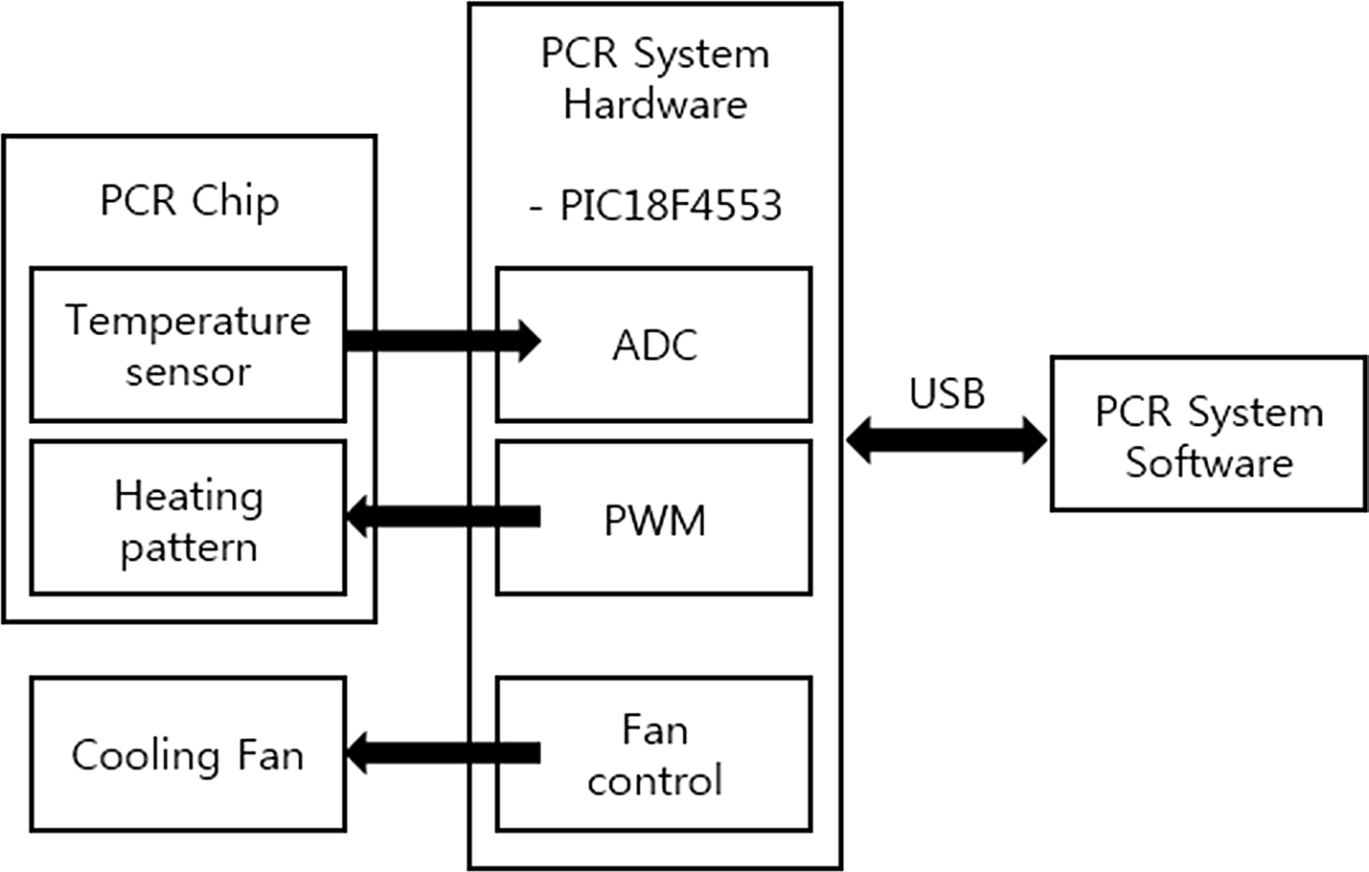
Overview of the proposed portable real-time PCR system

The proposed portable real-time PCR system has three primary components: the PCR chip, system hardware, and system software [9–11], as shown in Fig. 1. The PCR system hardware consists of the lens and detection module, LED module, integration board, micro-PCR chip (Microchip Technology’s PIC18F4553), and case.

The software delivers the proportional-integral-derivative (PID) value needed to maintain the hardware temperatures, and controls the cooling fan as needed to lower the temperature. The software also determines the temperature received from the hardware by using the delivered temperature value to track the protocol time of the relevant hardware.

The PCR system hardware receives the resistance value of the temperature sensor in the PCR chip from the analog-to-digital converter (ADC) and converts it into a temperature value which is then transferred to the software via a USB. The PCR chip temperature is maintained by delivering the value computed with the target temperature value (delivered from the software) and the PID (a variable for temperature control) via a pulse–width modulation (PWM) port. Then, the cooling fan uses a digital port to lower the chip temperature.

**Fig. 2:**
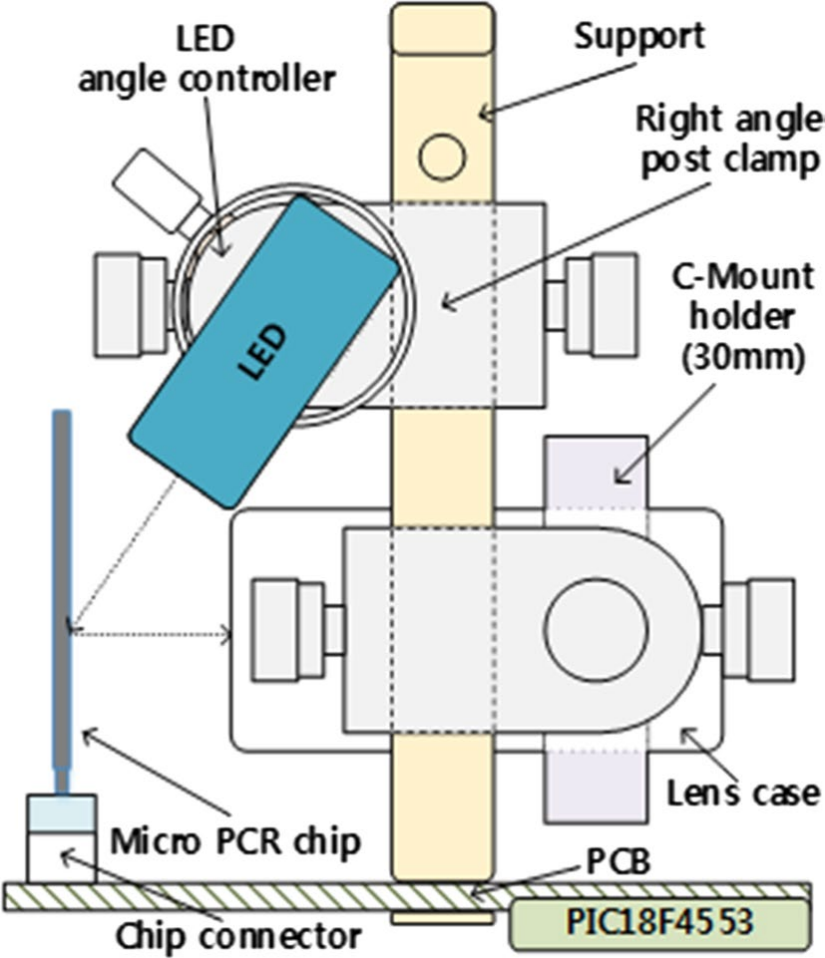
Side view of the proposed system

Figure 2 shows a detailed side view of the proposed system configuration. The system is composed of a case that blocks the light during fluorescence measurement, an integration board, a PIC board, an LED module, a micro-PCR chip, a lens module, and other parts that support and control each element. The LED module diagonally illuminates the light onto the micro-PCR chip, instead of using the typical orthogonal illumination.

The case, which was self-made to be the smallest size possible, blocks any outside light. The integration board (self-made PCB) and PIC board (Microchip Technology’s PIC18F4553) provide power by adjusting the power coming from the outside to correspond to that needed by the photodiode and LED. The LED module is equipped with a blue filter (excitation filter) and blue LED. The micro-PCR chip containing a reagent is exposed to the blue LED to make it fluorescent. The lens module includes a 24 mm ocular lens, an object lens, a green filter (emission filter), and a photodiode. It also measures the fluorescence of the reagent-containing chip.

**Fig. 3:**
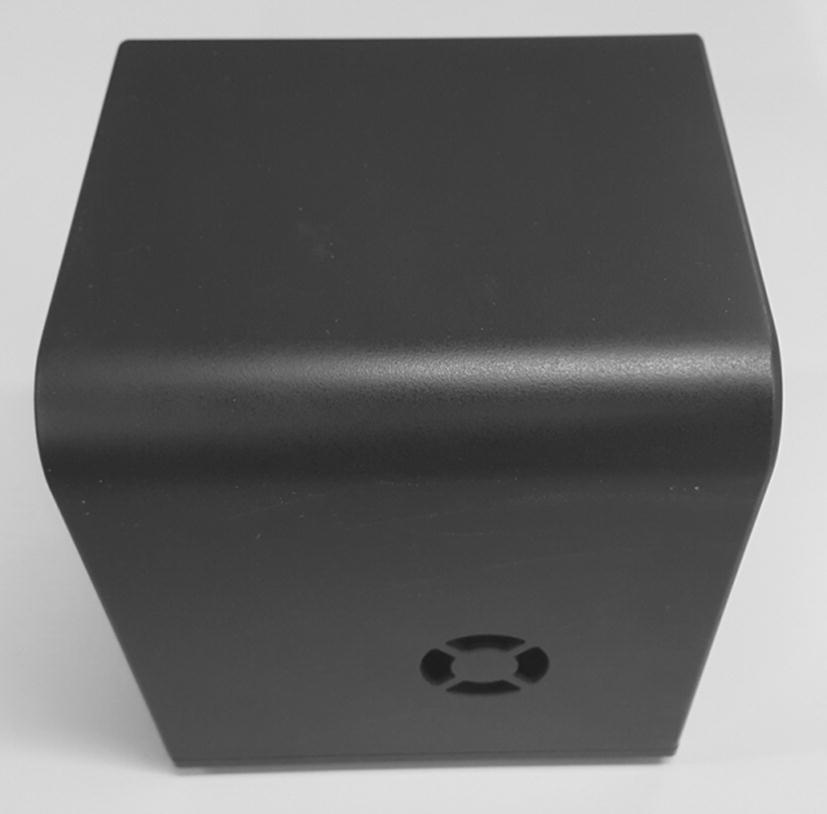
Actual image of the proposed system’s case

**Fig. 4:**
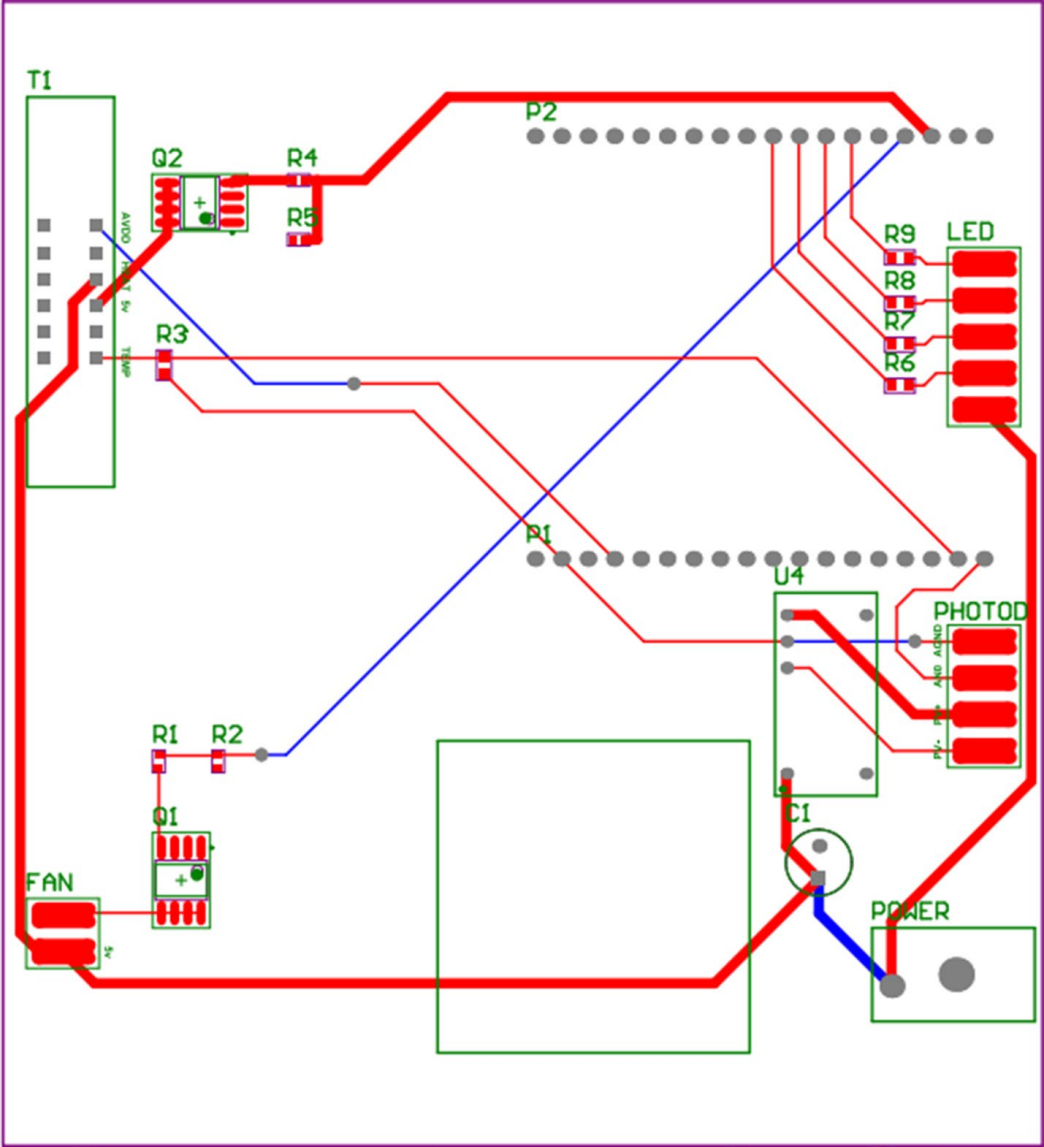
Integration board PCB design of the proposed system

Figure 3 shows an actual image of the system case used to block light. The case was fabricated to be the smallest size possible, and has dimensions 12.7 cm × 11 cm × 14 cm, which is much smaller than the existing system at 33 cm × 46 cm × 36 cm. The case has holes to allow power to be supplied to both the integration and PIC boards, and a fan hole to release heat. The bottom part of the case, where the integration board is installed, also has space for heat release. The integration board is composed of a fan to lower the chip temperature and a connector for inserting the chip. The board is also designed to allow a bar to be installed that fixes the placement of the LED and lens modules. The integration board can be assembled with the lower part of the case. Figure 4 illustrates the PCB design of the actual integration board operating the proposed system. The integration board supports each module and connects the electrical components.

**Fig. 5:**
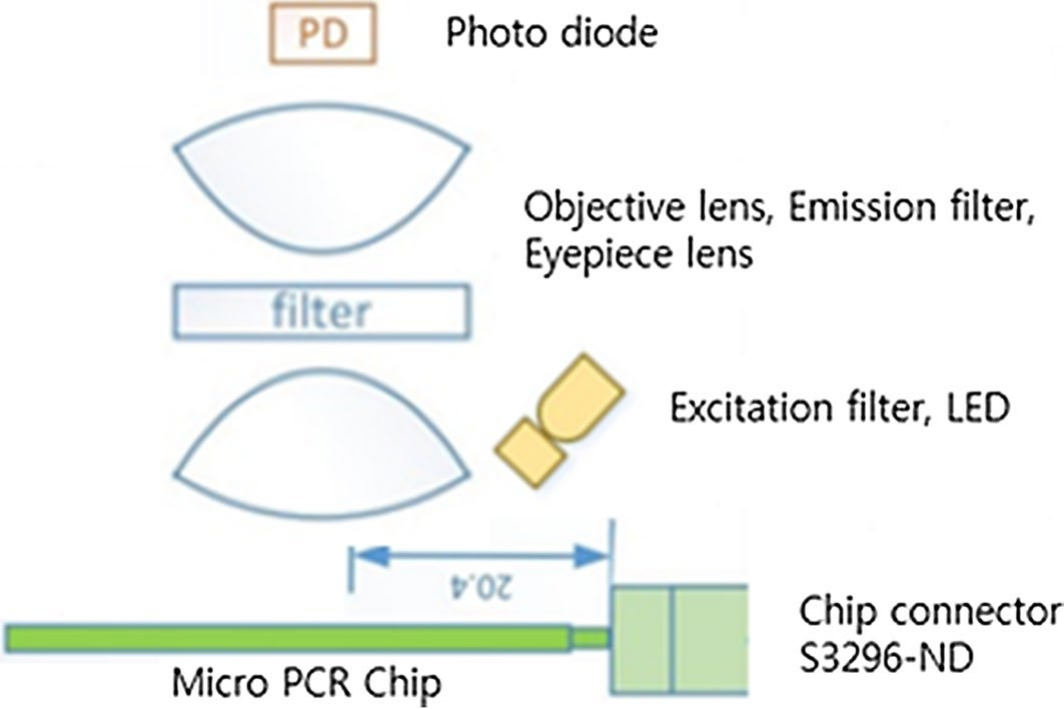
Composition and layout of the lens module

The lens module measures the reflected light by illuminating the blue LED onto the FAM fluorescent reagent inside the chip. The lens module consists of a 24 mm ocular lens, an object lens, a green filter (emission filter), and a photodiode. Figure 5 presents a detailed layout of the lens module. As seen in the figure, the order of the lens module from the bottom is as follows: micro-PCR chip, object lens, filter, ocular lens, and photodiode. The fluorescence inside the micro-PCR chip chamber receives light from the blue LED. The light passes the object lens, emission filter, and ocular lens, and then arrives at the photodiode. Its light energy is subsequently converted into electric energy, and the fluorescence brightness can be measured in real time. The side-illumination method was used when illuminating the blue LED light. Figure 5 also shows the lens module composition, including the micro-PCR chip for DNA amplification, fluorescent reagent, and connector for chip installation.

The filter used for the lens module is a commercially available, 25 mm emission filter, whereas that used for the LED module is a 12.5 mm excitation filter. The blue LED illuminates the FAM reagent inside the chip for fluorescence measurement. The LED in the LED module has a brightness of 9600 mcd, and it is commercially available (DigiKey Part No. 516-2275-1-ND). Table 1 shows the blue LED data sheet.

**Table 1:** Blue LED data sheet

Fluorochrome	DigiKey number	Wavelength	Luminous intensity	Voltage	Current test	Angle of field	Lens diameter
FAM	516-2275-1-ND	464 nm	9600 mcd	3.2 V	20 mA	15°	5 mm

The micro-controller unit (MCU), which is a processor used to exclusively control a specific system, is the core chip that functions as a brain in most electronic products. The MCU controls various product properties as a non-memory semiconductor. In this study, Microchip Technology’s PIC18F4553 was selected because it enables communication via USB ports.

**Fig. 6:**
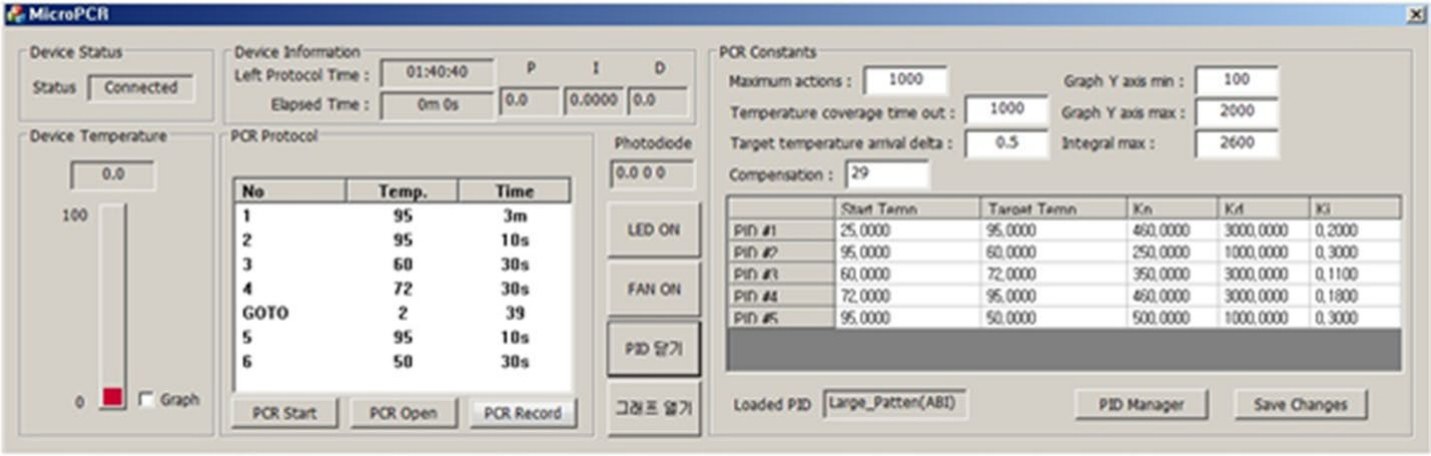
UI of the portable real-time PCR system software

**Fig. 7:**
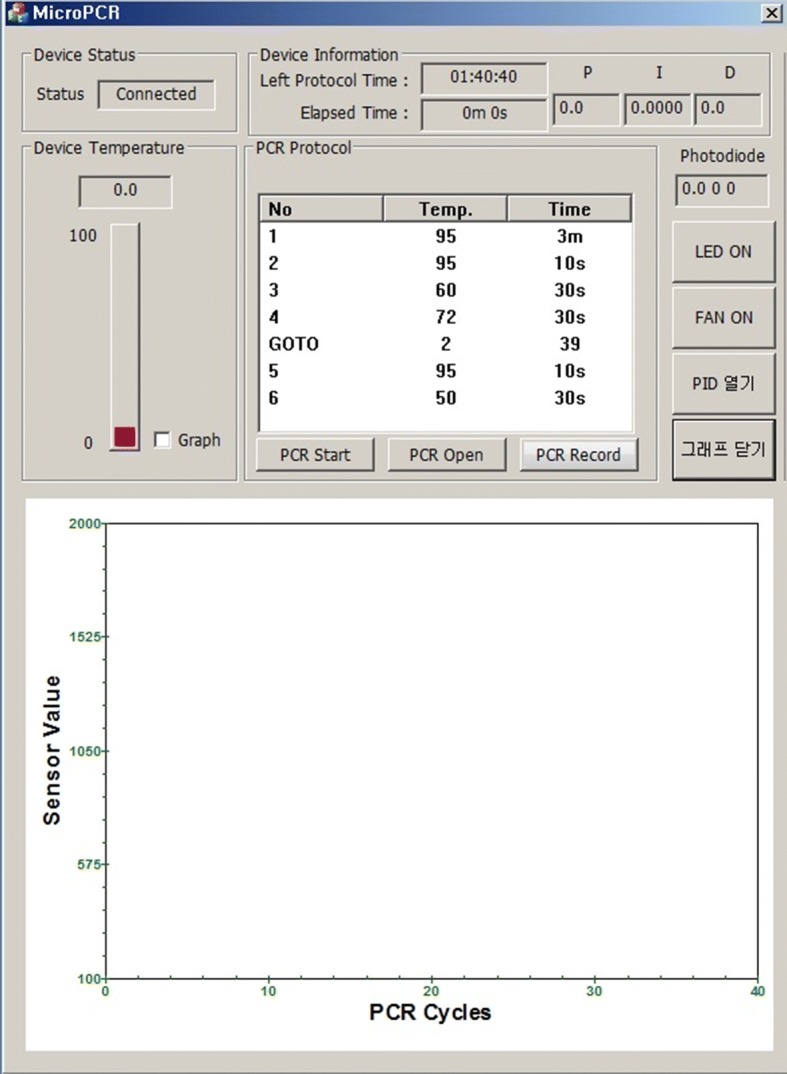
Main UI of the portable real-time PCR system software

The PCR system software is created using the Visual Studio 2012 MFC. The main function of the PCR system software is to perform the PCR protocol. We conducted our experiment using an independent PCR protocol, the PIDs of the inner temperature, and a chip temperature adequate for each reagent. The temperature for each section and the duration and number of cycles were written in the protocol. Figure 6 shows the PID value of the proposed system’s program and the user interface (UI) with an adjustable compensation value. Figure 7 displays the main UI prior to the PCR. The graph seen at the bottom of the main UI displays the fluorescence value measured in real time and the PCR cycles.

The temperature is the most important factor in PCR amplification. Accurate temperature measurement and control play core roles in shortening the analysis time and increasing the experimental accuracy. The inner temperature value is obtained by representing the linear regression line from the median of the target values (i.e., 65 °C, 72 °C, and 95 °C) and developing a formula for the slope. Therefore, an negative temperature coefficient (NTC) thermistor was attached to the center of the PCR chip chamber to measure the chip temperature during DNA amplification. However, it was necessary to measure the inner temperature of the PCR chip because this temperature differs from that of the reagent inside the chip. Accordingly, a commercially available thermocouple was used to confirm the actual reagent temperature inside the micro-PCR chip chamber. The 0.05 mm diameter thermocouple was a thin device without a sheath (CHAL-002, Omega Co., Ltd.). An additional reader was used (TC-08, Omega Co., Ltd.) because the thermocouple was not connected with a connector.

We measured the inner temperature using an internally implemented program with the library offered by the reader manufacturer. The optical module was removed and a USB microscope was used to check the chamber’s state in real time. The USB microscope replaces the optical module for experimental convenience during the calibration process. Furthermore, the PCR protocol for the calibration process was not used for the actual DNA amplification. A simplified protocol was utilized to measure the inner temperature. After measuring the temperature using a thermocouple, the result was compared to the NTC thermistor attached to the PCR chip. The temperature was then adjusted so that the reagent temperature inside the chamber can reach the target temperature of the PCR chip.

**Fig. 8:**
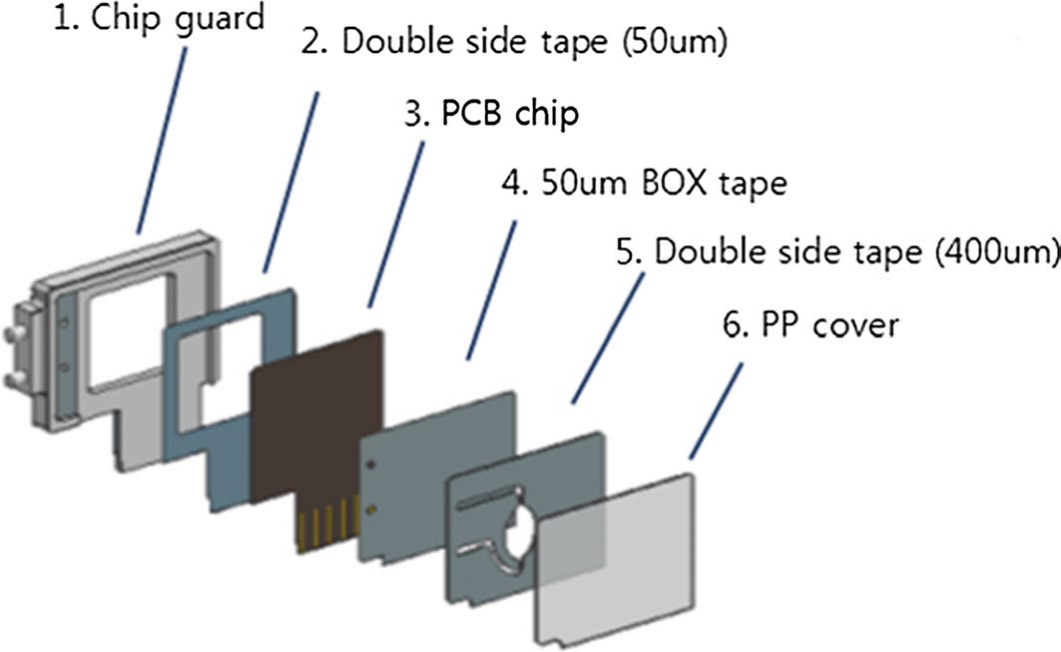
PCR chip structure

**Fig. 9:**
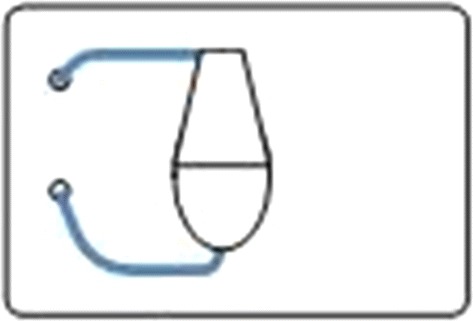
PCR chip chamber

**Fig. 10:**
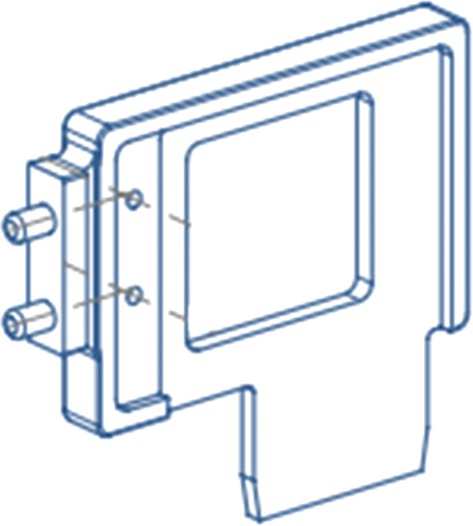
PCR chip housing

Figure 8 illustrates the system structure of the PCR chip used for DNA amplification. This structure was primarily composed of the PCR chip and the PCR chip housing that fixes the chip to prevent an error with the connector in the integration board. In this figure, the PP cover is made of 500 μm thick polypropylene. Double-sided tape that is 400 μm thick (Nitto’s 5620 BWN) is used. Accordingly, the 50 μm box tape is made of 50 μm thick 3 M 309 double-sided tape; the PCB chip is composed of a 200 μm thick black PCB; and double-sided tape (50 μm thick) is made of Nitto’s 5605 double-sided tape. Lastly, the chip guard functions as a guide that fixes the micro-PCR chip. Figures 9 and 10 illustrate the PCR chip chamber and the PCR chip housing, respectively. The size of the PCR chip chamber in Fig. 9 is 29.00 mm × 19.40 mm. The PCR chip housing in Fig. 10 measures 38.00 mm × 32.40 mm.

## Results

**Fig. 11:**
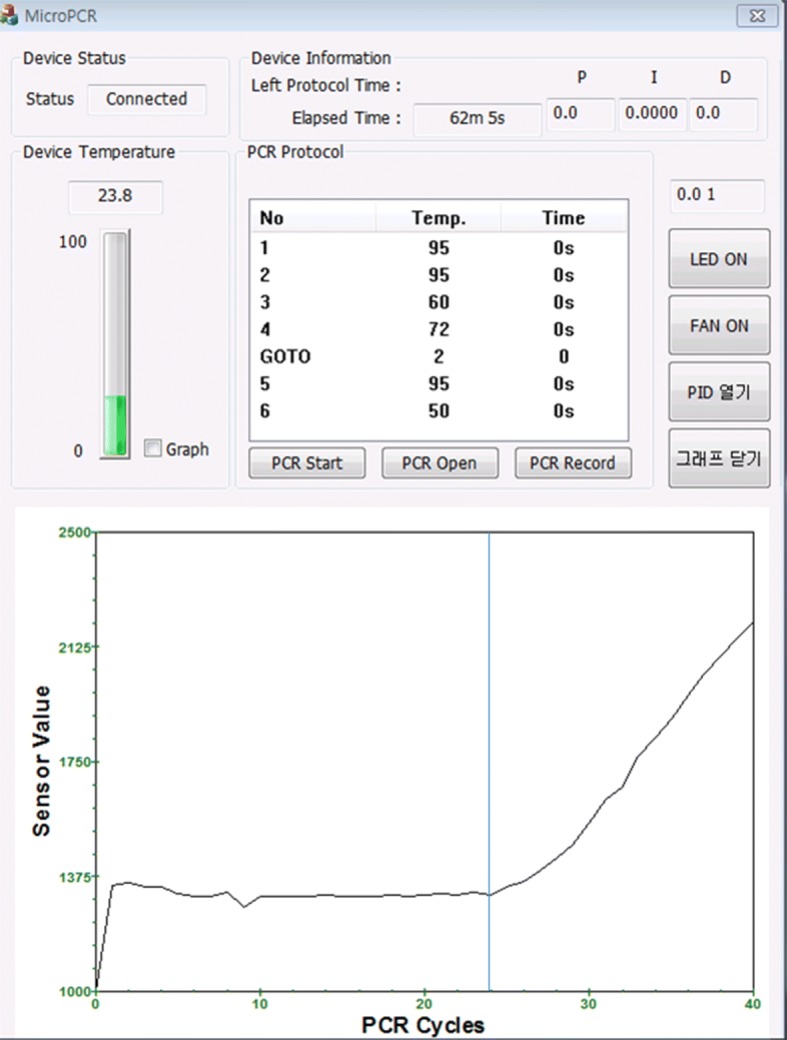
PCR result graph without the temperature adjustment

**Fig. 12:**
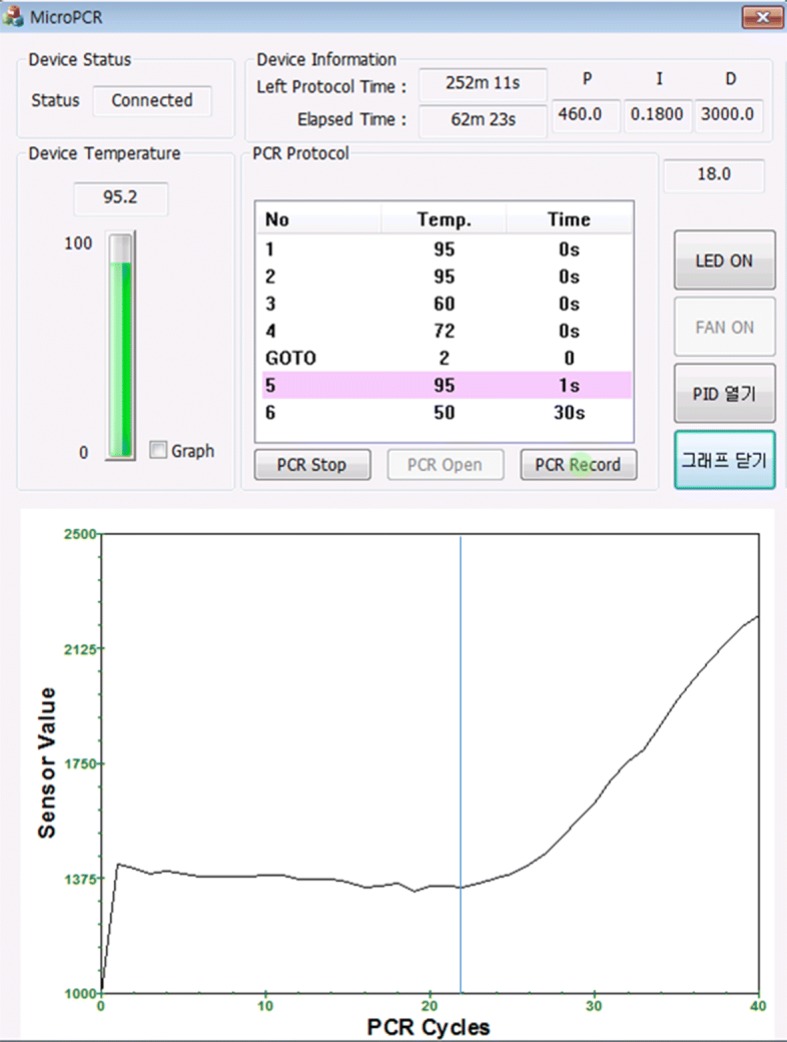
PCR result graph with the temperature adjustment

A PCR amplification experiment was conducted through a gene analysis using a small amount of DNA and a fluorescent reagent. The used DNA was for CT (Chlamydia trachomatis) and the fluorescence reagent was FAM (fluorescein amidite). The precise temperature of the reagent was subsequently calculated using the temperature adjustment coefficient. Figures 11 and 12 present the PCR amplification results without and with the temperature adjustment coefficient, respectively.

The x-axis represents the number of PCR amplification cycles. The y-axis denotes the fluorescent brightness of a reagent injected into the chamber, which was measured using a photodiode and converted into a voltage value with the mV unit. The actual study results after the application of the temperature adjustment parameter showed increasing values of early fluorescent brightness for the first PCR cycle. The point where the slope in the figure greatly increases is reduced from 24 to 22 cycles, indicating when the DNA amplification occurs.

## Discussion

In this study, we discovered that side illumination is more efficient than the conventional method. Side illumination can be used to reduce the size of real-time PCR devices. Thus, this system can replace existing PCR detection equipment, which is expensive, bulky, and difficult to move. Instead of using the typical illumination method, the side-illumination method is used to miniaturize the system’s optics. The most important factor in PCR amplification is the measurement of a small amount of DNA. Hence, an accurate measurement of the reagent temperature is necessary. A small difference in the reagent temperature can lead to increased experiment time and a higher error rate in the study results. Therefore, the temperature adjustment coefficient proposed in this study is used to derive a control parameter for the accurate modification of the reagent temperature. The coefficient usage ultimately increases the achievable accuracy in this study.

## Conclusions

A portable real-time PCR system was proposed in this work. The system was manufactured in-house, allowing a reduction in size compared with existing real-time PCR equipment. The portable system has the potential to replace current commercially available systems. It may lead to further decreases in manufacturing cost and permit the use of a simplified system while maintaining the same performance.

## Abbreviations

PCR: polymerase chain reaction
DNA: deoxyribonucleic acid
ADC: analog-to-digital converter
PWM: field-effect transistor
LED: light emitting diode
USB: universal serial bus
DSLR: digital single-lens reflex
NTC: negative temperature coefficient-thermic
PCB: printed circuit board
